# Erratum: The Role of Time as a Prognostic Factor in Pediatric Brain Tumors: a Multivariate Survival Analysis

**DOI:** 10.3389/pore.2022.1610756

**Published:** 2022-09-08

**Authors:** Pathology and Oncology Research Production Office

**Affiliations:** Frontiers Media SA, Lausanne, Switzerland

**Keywords:** Symptomatic interval, Delayed diagnosis, CNS tumors, Survival outcome, pretreatment interval

Due to a production error at the previous publisher, the Supplementary Material in the original article incorrectly included a copy of the manuscript.

The correct version of the Supplementary Material is published alongside this erratum.

The original version of this article has not been updated.

