# Supplementary material for: Erratum: The Role of Time as a Prognostic Factor in Pediatric Brain Tumors: a Multivariate Survival Analysis
Source: Pathol Oncol Res. 2022 Sep 8;28:1610756. doi: 10.3389/pore.2022.1610756 (PMC9496865; doi:10.3389/pore.2022.1610756)
Supplement: Supplementary file 1 [file DataSheet1.docx]

**Supporting Information**

**TABLE S1** Main categories of brain tumors and ICD-O3* Codes

| Name of brain tumor | ICD-O3* Codes |
| --- | --- |
| Astrocytoma, anaplastic | 9392/3 |
| Choroid plexus papilloma, NOS | 9390/0 |
| CNS Embryonal tumor with rhabdoid features | 9508/3 |
| Craniopharyngioma | 9350/1 |
| Diffuse midline glioma | 9385/3 |
| Diffuse astrocytoma | 9400/3 |
| Ependymoma, anaplastic | 9392/3 |
| Ependymoma, NOS | 9391/3 |
| Glioblastoma multiforme | 9440/3 |
| Medulloblastoma, classic | 9470/3 |
| Pilocytic astrocytoma | 9421/1 |
| Primitive neuroectodermal tumor, NOS | 9473/3 |
| Undifferentiated | NA |

* International Classification of Diseases for Oncology, 3rd Edition

**TABLE S2** Summary of patient in the main tumors

|  | Pilocytic Astrocytoma | Classic Medulloblastoma | Classic Ependymoma | Craniopharyngioma | Diffuse Midline Glioma | CNS ET/RF |
| --- | --- | --- | --- | --- | --- | --- |
| n | 36 | 23 | 12 | 9 | 15 | 8 |
| Sex = Male (%) | 21 (58.3) | 15 (65.2) | 5 (41.7) | 3 (33.3) | 9 (60.0) | 6 (75.0) |
| Age (median [IQR]) | 5.00 [4.00, 9.00] | 5.00 [2.50, 7.00] | 6.50 [4.75, 9.25] | 10.00 [9.00, 10.00] | 6.00 [5.00, 7.00] | 2.00 [1.75, 3.00] |
| Categorical age (%) |  |  |  |  |  |  |
| > 6 years | 14 (38.9) | 7 (30.4) | 6 (50.0) | 8 (88.9) | 6 (40.0) | 1 (12.5) |
| <=3 years | 7 (19.4) | 8 (34.8) | 1 (8.3) | 0 (0.0) | 3 (20.0) | 7 (87.5) |
| 3-6 years | 15 (41.7) | 8 (34.8) | 5 (41.7) | 1 (11.1) | 6 (40.0) | 0 (0.0) |
| Place of residency (%) |  |  |  |  |  |  |
| Mexico City | 18 (50.0) | 12 (52.2) | 5 (41.7) | 3 (33.3) | 5 (33.3) | 3 (37.5) |
| State of Mexico | 11 (30.6) | 5 (21.7) | 1 (8.3) | 3 (33.3) | 5 (33.3) | 4 (50.0) |
| Other areas | 7 (19.4) | 6 (26.1) | 6 (50.0) | 3 (33.3) | 5 (33.3) | 1 (12.5) |
| Survival time (median [IQR]) | 96.00 [81.00, 120.00] | 84.00 [48.00, 120.00] | 108.00 [93.00, 123.00] | 108.00 [72.00, 144.00] | 7.00 [5.00, 8.50] | 5.50 [3.50, 19.50] |
| Decease (%) | 5 (13.9) | 8 (34.8) | 1 (8.3) | 2 (22.2) | 12 (80.0) | 7 (87.5) |
| Localization = Supratentorial (%) | 11 (30.6) | 0 (0.0) | 5 (41.7) | 9 (100.0) | 0 (0.0) | 2 (25.0) |
| Tumor Grade = High (%) | 0 (0.0) | 23 (100.0) | 0 (0.0) | 0 (0.0) | 15 (100.0) | 8 (100.0) |
| Type of Surgery (%) |  |  |  |  |  |  |
| Total resection | 13 (36.1) | 8 (34.8) | 8 (66.7) | 3 (33.3) | 0 (0.0) | 0 (0.0) |
| Biopsy | 8 (22.2) | 0 (0.0) | 0 (0.0) | 1 (11.1) | 1 (6.7) | 2 (25.0) |
| None | 1 (2.8) | 0 (0.0) | 0 (0.0) | 1 (11.1) | 14 (93.3) | 0 (0.0) |
| Partial resection | 14 (38.9) | 15 (65.2) | 4 (33.3) | 4 (44.4) | 0 (0.0) | 6 (75.0) |
| Coadjuvant treatments (%) |  |  |  |  |  |  |
| Both | 16 (44.4) | 20 (87.0) | 3 (25.0) | 8 (88.9) | 15 (100.0) | 4 (50.0) |
| Chemotherapy alone | 1 (2.8) | 0 (0.0) | 0 (0.0) | 0 (0.0) | 0 (0.0) | 2 (25.0) |
| None | 14 (38.9) | 1 (4.3) | 1 (8.3) | 0 (0.0) | 0 (0.0) | 2 (25.0) |
| Radiation therapy alone | 5 (13.9) | 2 (8.7) | 8 (66.7) | 1 (11.1) | 0 (0.0) | 0 (0.0) |
| Prediagnostic symptomatic intervals [days] (median [IQR]) | 90.00 [60.00, 127.50] | 90.00 [45.00, 150.00] | 105.00 [60.00, 180.00] | 210.00 [90.00, 270.00] | 120.00 [60.00, 150.00] | 90.00 [26.25, 135.00] |
| Prediagnostic symptomatic intervals [categorical] (%) (%) | |  |  |  |  |  |
| <=3 months | 19 (52.8) | 14 (60.9) | 6 (50.0) | 3 (33.3) | 7 (46.7) | 4 (50.0) |
| 3-6 months | 11 (30.6) | 5 (21.7) | 5 (41.7) | 1 (11.1) | 7 (46.7) | 3 (37.5) |
| >6 months | 6 (16.7) | 4 (17.4) | 1 (8.3) | 5 (55.6) | 1 (6.7) | 1 (12.5) |
| Pre treatment interval [days] (median [IQR]) | 12.00 [8.00, 15.00] | 14.00 [10.00, 17.50] | 12.50 [8.50, 18.00] | 18.00 [13.00, 30.00] | 12.00 [9.00, 20.00] | 10.00 [8.00, 11.75] |
| Pre treatment interval [categorical] = >13 days (%) | 11 (30.6) | 12 (52.2) | 6 (50.0) | 5 (55.6) | 7 (46.7) | 2 (25.0) |
| Global delay interval [days] (median [IQR]) | 109.00 [66.75, 145.75] | 100.00 [57.50, 171.00] | 117.50 [68.25, 198.00] | 220.00 [108.00, 330.00] | 132.00 [68.00, 165.00] | 102.50 [29.50, 143.50] |
| Global delay interval [categorical] (%) | |  |  |  |  |  |
| <=3m | 15 (41.7) | 9 (39.1) | 4 (33.3) | 0 (0.0) | 5 (33.3) | 4 (50.0) |
| 3-6m | 13 (36.1) | 9 (39.1) | 4 (33.3) | 3 (33.3) | 9 (60.0) | 2 (25.0) |
| >6m | 8 (22.2) | 5 (21.7) | 4 (33.3) | 6 (66.7) | 1 (6.7) | 2 (25.0) |

**TABLE S3** Summary of patient in the category others

|  | Anaplastic Ependymoma | Primitive Neuroectodermal Tumor | Diffuse Astroctytoma | Glioblastoma multiforme | Anaplastic Astrocytoma | Choroid Plexus Papilloma | Undifferentiated |
| --- | --- | --- | --- | --- | --- | --- | --- |
| n | 4 | 5 | 3 | 2 | 2 | 3 | 5 |
| Sex = Male (%) | 0 (0.0) | 1 (20.0) | 2 (66.7) | 0 (0.0) | 1 (50.0) | 2 (66.7) | 3 (60.0) |
| Age (median [IQR]) | 4.00 [3.75, 4.00] | 3.00 [3.00, 5.00] | 6.00 [3.50, 7.00] | 9.50 [8.75, 10.25] | 7.50 [7.25, 7.75] | 2.00 [2.00, 3.00] | 7.00 [5.00, 9.00] |
| Categorical age (%) |  |  |  |  |  |  |  |
| > 6 years | 0 (0.0) | 0 (0.0) | 1 (33.3) | 2 (100.0) | 2 (100.0) | 0 (0.0) | 3 (60.0) |
| <=3 years | 1 (25.0) | 3 (60.0) | 1 (33.3) | 0 (0.0) | 0 (0.0) | 2 (66.7) | 0 (0.0) |
| 3-6 years | 3 (75.0) | 2 (40.0) | 1 (33.3) | 0 (0.0) | 0 (0.0) | 1 (33.3) | 2 (40.0) |
| Place of residency (%) |  |  |  |  |  |  |  |
| Mexico City | 3 (75.0) | 2 (40.0) | 1 (33.3) | 0 (0.0) | 0 (0.0) | 2 (66.7) | 1 (20.0) |
| State of Mexico | 1 (25.0) | 3 (60.0) | 2 (66.7) | 1 (50.0) | 1 (50.0) | 1 (33.3) | 2 (40.0) |
| Other areas | 0 (0.0) | 0 (0.0) | 0 (0.0) | 1 (50.0) | 1 (50.0) | 0 (0.0) | 2 (40.0) |
| Survival time (median [IQR]) | 108.00 [82.75, 111.00] | 96.00 [12.00, 96.00] | 108.00 [66.00, 126.00] | 60.00 [42.00, 78.00] | 66.00 [39.00, 93.00] | 84.00 [78.00, 90.00] | 96.00 [96.00, 120.00] |
| Decease (%) | 1 (25.0) | 2 (40.0) | 1 (33.3) | 1 (50.0) | 0 (0.0) | 0 (0.0) | 0 (0.0) |
| Localization = Supratentorial (%) | 3 (75.0) | 5 (100.0) | 2 (66.7) | 2 (100.0) | 1 (50.0) | 1 (33.3) | 5 (100.0) |
| Tumor Grade = High (%) | 4 (100.0) | 5 (100.0) | 0 (0.0) | 2 (100.0) | 2 (100.0) | 0 (0.0) | 1 (20.0) |
| Type of Surgery (%) |  |  |  |  |  |  |  |
| Total resection | 0 (0.0) | 2 (40.0) | 1 (33.3) | 1 (50.0) | 1 (50.0) | 1 (33.3) | 2 (40.0) |
| Biopsy | 0 (0.0) | 0 (0.0) | 0 (0.0) | 0 (0.0) | 1 (50.0) | 0 (0.0) | 0 (0.0) |
| None | 0 (0.0) | 0 (0.0) | 1 (33.3) | 0 (0.0) | 0 (0.0) | 0 (0.0) | 0 (0.0) |
| Partial resection | 4 (100.0) | 3 (60.0) | 1 (33.3) | 1 (50.0) | 0 (0.0) | 2 (66.7) | 3 (60.0) |
| Coadjuvant treatments (%) |  |  |  |  |  |  |  |
| Both | 4 (100.0) | 3 (60.0) | 2 (66.7) | 2 (100.0) | 2 (100.0) | 0 (0.0) | 1 (20.0) |
| Chemotherapy alone | 0 (0.0) | 1 (20.0) | 1 (33.3) | 0 (0.0) | 0 (0.0) | 0 (0.0) | 0 (0.0) |
| None | 0 (0.0) | 0 (0.0) | 0 (0.0) | 0 (0.0) | 0 (0.0) | 0 (0.0) | 3 (60.0) |
| Radiation therapy alone | 0 (0.0) | 1 (20.0) | 0 (0.0) | 0 (0.0) | 0 (0.0) | 3 (100.0) | 1 (20.0) |
| Prediagnostic symptomatic intervals [days] (median [IQR]) | 180.00 [165.00, 180.00] | 90.00 [60.00, 120.00] | 150.00 [120.00, 165.00] | 135.00 [127.50, 142.50] | 45.00 [37.50, 52.50] | 60.00 [45.00, 390.00] | 90.00 [90.00, 1440.00] |
| Prediagnostic symptomatic intervals [categorical] (%) (%) |  |  |  |  |  |  |  |
| <=3 months | 0 (0.0) | 3 (60.0) | 1 (33.3) | 0 (0.0) | 2 (100.0) | 2 (66.7) | 3 (60.0) |
| 3-6 months | 4 (100.0) | 1 (20.0) | 2 (66.7) | 2 (100.0) | 0 (0.0) | 0 (0.0) | 0 (0.0) |
| >6 months | 0 (0.0) | 1 (20.0) | 0 (0.0) | 0 (0.0) | 0 (0.0) | 1 (33.3) | 2 (40.0) |
| Pre treatment interval [days] (median [IQR]) | 60.00 [50.00, 60.00] | 15.00 [12.00, 20.00] | 15.00 [15.00, 17.50] | 17.50 [16.25, 18.75] | 9.00 [8.50, 9.50] | 30.00 [25.00, 30.00] | 18.00 [14.00, 18.00] |
| Pre treatment interval [categorical] = >13 days (%) | 4 (100.0) | 3 (60.0) | 3 (100.0) | 2 (100.0) | 0 (0.0) | 3 (100.0) | 4 (80.0) |
| Global delay interval [days] (median [IQR]) | 240.00 [215.00, 240.00] | 120.00 [75.00, 132.00] | 165.00 [135.00, 182.50] | 152.50 [143.75, 161.25] | 54.00 [46.00, 62.00] | 90.00 [70.00, 420.00] | 108.00 [108.00, 1445.00] |
| Global delay interval [categorical] (%) |  |  |  |  |  |  |  |
| <=3m | 0 (0.0) | 2 (40.0) | 0 (0.0) | 0 (0.0) | 2 (100.0) | 2 (66.7) | 1 (20.0) |
| 3-6m | 1 (25.0) | 2 (40.0) | 2 (66.7) | 2 (100.0) | 0 (0.0) | 0 (0.0) | 2 (40.0) |
| >6m | 3 (75.0) | 1 (20.0) | 1 (33.3) | 0 (0.0) | 0 (0.0) | 1 (33.3) | 2 (40.0) |
